# Autophagic responses to vigorous endurance exercise in men vary by training status but are similar in PBMCs and skeletal muscle: A pilot study

**DOI:** 10.14814/phy2.71064

**Published:** 2026-08-19

**Authors:** Jonathan W. Specht, Jeremy B. Ducharme, Alyssa R. Bailly, Michael R. Deyhle

**Affiliations:** ^1^ Department of Health, Exercise and Sports Sciences University of New Mexico Albuquerque New Mexico USA; ^2^ Department of Kinesiology & Athletic Training University of Northern Iowa Cedar Falls Iowa USA; ^3^ Department of Cell Biology and Physiology, School of Medicine University of New Mexico Albuquerque New Mexico USA

**Keywords:** autophagy, exercise, LC3II, p62, trained, untrained

## Abstract

Vigorous exercise triggers signaling cascades that activate autophagy markers like the degradation of sequestosome 1 (p62) and accumulation of Light Chain 3 II (LC3II). Limited human data exist on autophagic responses across different local and systemic tissues and how training status affects these relationships. This study investigates the vigorous exercise‐induced changes in p62 and LC3II expression in peripheral blood mononuclear cells (PBMCs) and skeletal muscle between endurance‐trained and untrained men. Twelve men (endurance‐trained *n* = 7, untrained *n* = 5) completed 60 min of cycling at their second ventilatory threshold. Skeletal muscle biopsy samples and PBMCs were collected pre‐ and 3‐h post‐exercise and analyzed for p62 and LC3II protein expression. We found significant interaction effects of time and training status for p62 (*p* < 0.001) and LC3II (*p* = 0.002). In untrained men, p62 decreased in PBMCs (FC = 0.50 ± 0.14; *p* < 0.001) and skeletal muscle (FC = 0.57 ± 0.21; *p* < 0.001), while LC3II increased in both tissues (FC = 1.74 ± 0.79; *p* = 0.019 for PBMCs; FC = 1.69 ± 0.47; *p* = 0.033 for skeletal muscle). No changes were observed in endurance‐trained men (all *p* > 0.05). These results suggest that a bout of vigorous endurance exercise increased autophagy‐related markers in both skeletal muscle and PBMCs in the untrained men only, suggesting a diminished autophagic response in the trained men.

## Graphical abstract

Training status modulates autophagic responses to endurance exercise. LC3II: Light Chain 3 II; p62: sequestosome 1.

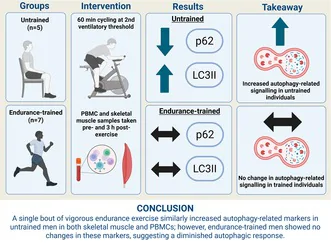

## 1 INTRODUCTION

Acute exercise imposes physiological strain that can induce biological adaptations, enhancing physical capacities over time by local (e.g., skeletal muscle) and systemic (e.g., peripheral blood mononuclear cells [PBMCs]) activation of stress response mechanisms. Specifically, immune, hormonal, metabolic, and hypoxic pathways are upregulated to allow the body to adapt to fluctuating energy demands and maintain homeostasis (Contrepois et al., 2020). Autophagy, an evolutionary conserved cellular recycling process, is an additional mechanism which growing evidence supports as a large contributing factor to exercise‐induced adaptations (Halling & Pilegaard, 2017). Indeed, exercise‐induced autophagy has been associated with improved mitochondrial function and glucose metabolism, cardiac remodeling, changes in muscle mass, and creation of new blood vessels (i.e., angiogenesis) (Ferraro et al., 2014; He, Bassik, et al., 2012; Ju et al., 2016; Lira et al., 2013).

Upregulation of autophagy involves a cascade of cellular signaling, often assessed by observing hallmark alterations in markers such as the conversion of microtubule‐associated protein 1A/1B‐light chain 3‐I (LC3I) to LC3II, reflecting autophagosome formation, and the degradation of sequestosome‐1 (p62), a protein that binds damaged proteins and is cleared during autophagy (Liu et al., 2016; Tanida et al., 2008). Both of which have been observed in local skeletal muscle and systemic PBMCs following acute exercise (Escobar et al., 2021; McCormick et al., 2022). While transient activation of autophagy is essential for maintaining cellular homeostasis and contributes to beneficial exercise adaptations, dysfunctional autophagic activity, whether excessive or insufficient, has been implicated in various systemic (e.g., cancer, type 2 diabetes, and Alzheimer's) and skeletal muscle (e.g., Danon disease, Duchenne muscular dystrophy, and sarcopenia) pathologies (Li et al., 2017; Mathew et al., 2007; Xia et al., 2021; Yang et al., 2017), highlighting the importance of the timing, duration, and extent of autophagic activity.

In regards to exercise, the stimuli responsible for initiating beneficial processes are multifactorial, and previous research has shown that acute autophagic responses to exercise depend on factors such as intensity, duration, and modality of exercise (Zhou et al., 2025; Halling & Pilegaard, 2017). While much of the existing literature has focused on systemic activation of autophagy, primarily measured in PBMCs (Escobar et al., 2021), there has been limited attention given to potential tissue‐specific variations in autophagic activity following acute exercise. Understanding the tissue‐specific autophagic responses of skeletal muscle compared to systemic PBMC following exercise is valuable to further understand the muscle specific remodeling processes, which are important for training adaptation, recovery, and preventing muscle dysfunction. To our knowledge, only one prior study has directly compared autophagic marker responses between skeletal muscle and PBMCs following acute exercise, as Escobar et al. (2021) observed distinct differences in the regulation of autophagic markers between PBMCs and skeletal muscle following high‐intensity versus moderate‐intensity exercise. This finding indicates that variable training strategies may be needed to target local versus systemic exercise adaptations. For example, regulation of PBMCs may be targeted in cases of chronic inflammation, immunosenescence, and autoimmune disease, whereas targeting skeletal muscle‐specific autophagy may be beneficial regarding training adaptations, skeletal muscle disorders, and sarcopenia. Despite these findings, much remains unknown about how endurance training might modulate the acute autophagic response across different tissues, including PBMCs and skeletal muscle, and how these responses may differ between trained and untrained populations.

Herein, the current study aims to address this gap in the literature by determining the extent to which training status (endurance‐trained vs. untrained) and tissue type (PBMCs vs. skeletal muscle) influence the expression of markers of autophagy in men following an acute one‐hour bout of vigorous endurance exercise. As a secondary analysis from a previous project, this study also aims to provide a foundation regarding hypotheses and power calculations for future investigations.

## 2 METHODS

This project is a secondary analyses from a previous investigation of the effect of training status on the regulation of Toll‐like receptor 4 expression in muscle and PBMCs following vigorous endurance exercise (Ducharme et al., 2025). Briefly, 12 endurance‐trained (*n* = 7) and untrained (*n* = 5) men who had blood and muscle samples taken during the original protocol were included in this study. While the primary study examined Toll‐like receptor 4 signaling, which can act upstream of multiple inflammatory pathways, autophagy represents a parallel and functionally distinct cellular program with its own mechanistic and clinical relevance. This analysis was therefore conducted to provide novel insight into training‐dependent autophagic responses across tissue compartments. Inclusion criteria included no use of medications affecting exercise‐induced immune responses (e.g., NSAIDs, glutamine, and probiotics), no diseases impacting inflammation (e.g., diabetes, HIV/AIDS, arthritis, and cardiovascular disease), and no tobacco use within 6 months. They arrived euhydrated (urine specific gravity ≤1.020, measured manually with a refractometer) after an overnight fast. All participants provided signed informed consent following verbal and written explanation of the study. All procedures were approved by the University of New Mexico Institutional Review Board (Protocol Number: 2209015849) and were conducted in accordance with the *Declaration of Helsinki*, with the exception of registration in a database.

### 2.1 Experimental design

During the first of two visits, participants answered health and physical activity questionnaires, and completed baseline assessments (height, body mass, body fat percentage, VO_2_peak, and second ventilatory threshold; VT2). Men who reported ≥300 weekly minutes of moderate‐intensity endurance exercise over the past 6 months were classified as endurance‐trained, and those reporting <150 min weekly and no vigorous exercise were deemed untrained. Participants returned for the experimental trial at least 48 h later, where they completed a 1‐h cycling session at an intensity corresponding to their VT2. Blood and skeletal muscle samples were collected pre‐ and 3 h post‐exercise. Experimental trials took place in the morning between 06:00 and 10:00 after an overnight fast to reduce the impact of diurnal variation or nutrition.

Height was measured with no shoes on using a stadiometer (seca, Hamburg, Germany), while body mass and percent body fat were assessed while barefoot using a segmental multi‐frequency InBody 770 analyzer (InBody, Cerritos, CA). Following a 5‐min warm‐up on a cycle ergometer (Excalibur Sport, Lode Medical Technology, Netherlands) at a self‐selected intensity, participants completed a maximal exercise cycling test using a ramp protocol (20–35 watts/min) until they reached volitional exhaustion. The intensity progression of the protocol was individualized based on body mass and activity level so that exhaustion was reached in 8–12 min (Yoon et al., 2007). Expired air was collected and analyzed by a metabolic cart (ParvoMedics TrueOne 2400, UT), which was calibrated according to manufacturer guidelines prior to testing. The highest VO_2_ value averaged over 8 breaths was recorded as VO_2_peak to minimize outliers as previously described (Ducharme et al., 2022). Using the V‐slope method (Beaver et al., 1986), two independent researchers identified the intensity corresponding to the VT2, which was used for the following trial. During the experimental trial, participants cycled for 1 h at their VT2 workload. Watts and rating of perceived exertion (RPE; 6–20 scale) were recorded every 5 min and averaged over the session. If RPE exceeded 17, the workload for the subsequent 5‐min bout was reduced by 5 watts to ensure participants completed 1 h of vigorous exercise.

### 2.2 Blood sampling

Venous blood samples were collected through venipuncture of an antecubital vein into vacutainers containing EDTA (BD Biosciences, Franklin Lakes, NJ) pre‐ and 3‐h post‐exercise. Whole blood from EDTA vacutainers was then layered over Histopaque 1077 (Sigma Aldrich, St. Louis, MO) in a 1:1 ratio (15 mL/15 mL) and centrifuged at 960 × g for 20 min. The buffy coat containing PBMCs was then collected, transferred to a clean conical centrifuge tube, suspended with 10 mL of phosphate buffered saline (PBS), and centrifuged at 600 × g for 10 min. The supernatant was removed, and the cell pellet containing PBMCs was stored at −80°C for subsequent analyses.

### 2.3 Skeletal muscle tissue sampling

Samples of whole skeletal muscle were obtained from the dominant leg of participants' *m. vastus lateralis* (VL) pre‐ and 3‐h post‐exercise. Prior to muscle biopsy, participants' limb was sanitized with sterile alcohol swabs, antiseptic (iodine), followed by superficial and deep injection of approximately 3 cc of local anesthesia (Lidocaine, 2% without epinephrine). Approximately 20 mg of skeletal muscle was sampled from the VL of each participant's dominant leg using a 14‐gauge micro‐biopsy needle (Argon Medical Devices, Frisco, TX). For 3‐h post‐muscle biopsies, the VL was identified, and a target location was selected just proximal (within a few millimeters) to the initial site to ensure comparable tissue samples while minimizing damage to any single area, which has been shown to reduce biopsy artifact (Van Thienen et al., 2014). Collected tissue samples were cleaned of debris, washed in ice‐cold PBS, and separated for future immunoblotting. Samples were then flash frozen in liquid nitrogen and stored at −80°C.

### 2.4 Immunoblotting

PBMC samples were lysed with 200 μL of ice‐cold RIPA commercial lysis buffer including Halt protease inhibitor cocktail and phosphatase inhibitor cocktail (ThermoFisher Scientific, 78446) before being centrifuged at 10000 × *g* for 10 min at 4°C. Muscle samples were homogenized using a bead homogenizer (Beadbug 3, Benchmark Scientific, Sayrenville, NJ) with approximately 300 μL of ice‐cold RIPA commercial lysis buffer including Halt protease inhibitor cocktail and phosphatase inhibitor cocktail (ThermoFisher Scientific) before also being centrifuged at 10000 × *g* for 10 min at 4°C. The supernatant for each sample was stored at −80°C for further analysis. Total protein content of each sample was measured using a protein assay kit (Pierce BCA, ThermoFisher Scientific, 23225). A 4X Laemmli buffer with 5% β‐mercaptoethanol was added to samples and incubated at 100°C for 10 min for use in gel loading. Thirty μg of protein was added to each lane and separated by electrophoresis on precast 4%–20% polyacrylamide gels (BioRad) and transferred to polyvinylidene difluoride membranes (Sigma Aldrich, P2938) via Trans‐blot Turbo Transfer (BioRad). Membranes were blocked for 90 min in 5% dry milk in Tris buffered saline plus 0.05% Tween 20 buffer solution (TBST). Membranes were cut according to the molecular weight of the protein of interest. Membranes were incubated overnight at 4°C with primary antibodies for rabbit polyclonal SQSRM1/p62 (1:1000, Cat. No. 5114, Cell Signaling Technology, RRID:AB_10624872) and rabbit polyclonal LC3B (1:1000, Cat. No. 2775, Cell Signaling Technology, RRID:AB_915950). Following overnight incubation, membranes were washed with TBST and incubated with respective horseradish peroxidase‐conjugated secondary antibodies purchased from Cell Signaling Technology against rabbit (1:2000, Cat. No. 7074, RRID:AB_2099233) in 5% dry milk in TBST for 1‐h at room temperature followed by an additional set of washes in TBST. Membranes were then incubated in a chemiluminescent solution for 3 min before being imaged with a ChemiDoc Touch Imaging System (BioRad). The chemiluminescence reagent was made with 2 mmol/L 4‐iodophenylboronic acid (Sigma, 471933), 1.25 mmol/L luminol (Sigma Aldrich, 123072), 5.3 mmol/L hydrogen peroxide (Sigma Aldrich, 216763) in 100 mmol/L Tris/HCl pH 8.8 (Invitrogen, 15568025) (Haan & Behrmann, 2007). Image Lab software (Version 6.0.1, BioRad) was used to quantify protein expressions. LC3B detects endogenous levels of total LC3B protein however stronger reactivity is observed with the type II form of LC3B (cell signaling/manufacturer). Therefore, in the current study wherein only one band was detected at the expected molecular weight of LC3II (~14–16 kDa), LC3B signal was treated as a proxy for LC3II. The absence of a visible LC3I band may reflect low LC3I abundance under these experimental conditions, consistent with its rapid conversion to LC3II during active autophagy (Tanida et al., 2008). All proteins of interest were normalized to total protein via Ponceau S Stain (Cell Signaling Technology) and expressed as relative fold change (FC) from corresponding pre‐exercise time points on each gel serving as baseline.

### 2.5 Statistical analyses

A three‐way mixed model ANOVA (with main effects of tissue type, training status, and time). Time (pre‐ vs. 3 h post‐exercise) and tissue type (PBMCs vs. skeletal muscle) were analyzed as within‐subject repeated measures, as each participant provided both tissue samples at each time point, and training status (endurance‐trained vs. untrained) was analyzed as a between‐subject factor. To determine differences among the protein and gene expression of p62 and LC3II protein in skeletal muscle and PBMCs pre‐ to 3‐h post‐exercise between trained and untrained men, Tukey's honestly significant difference (HSD) post hoc test testing was conducted if there was a significant time × training interaction effect. Pearson's or Spearman's correlations analyses, depending on the normality of the outcomes assessed, along with simple linear regression analyses were used to determine relationships between changes in p62 and LC3II protein expression in both PBMCs and skeletal muscle samples. Two‐tailed paired *t*‐tests were conducted to compare characteristics of trained and untrained individuals. Statistical analyses were performed using GraphPad Prism 10.3 (GraphPad Software, Inc., La Jolla, CA). When applicable, data are reported as mean ± standard deviation and effect sizes are presented as partial eta‐squared ($\eta_{p}^{2}$). Statistical significance was set a priori to *p* ≤ 0.05.

## 3 RESULTS

### 3.1 Participant characteristics and physiological responses to the experimental trial

Characteristics for the 12 participants who were screened and completed this study have been reported elsewhere (Ducharme et al., 2025). Briefly, there were no differences in age (Untrained: 24.2 ± 5.4 years; Endurance‐trained: 25.0 ± 5.4 years; *p* = 0.823), height (Untrained: 183.1 ± 6.9 cm; Endurance‐trained: 183.5 ± 5.0 cm; *p* = 0.919), body fat percentage (Untrained: 14.8% ± 5.3%; Endurance‐trained: 11.4% ± 4.4%; *p* = 0.242), or body mass (Untrained: 81.2 ± 11.5 kg; Endurance‐trained: 77.5 ± 11.4 kg; *p* = 0.480) between groups. However, as expected, endurance‐trained participants had significantly higher VO_2_peaks (Untrained: 38.2 ± 2.5 mL/kg/min; Endurance‐trained: 54.3 ± 4.4 mL/kg/min; *p* < 0.001) and maximum workloads (Untrained: 294 ± 41 W; Endurance‐trained: 381 ± 38 W; *p* = 0.001), but no difference in maximum heart rates (*p* = 0.237) compared to untrained participants. During the experimental trial, the initial workload was maintained for most (*n* = 5 endurance‐trained; *n* = 3 untrained), and all participants completed the entire 1‐h of vigorous endurance exercise. Workload was decreased by a maximum of 20 watts for participants who needed a reduced effort to stay near their VT2 and complete the hour based on monitored feedback during the test (e.g., HR and RPE). As reported elsewhere (Ducharme et al., 2025), endurance‐trained individuals had higher ventilatory thresholds (*p* = 0.004) and exercised at greater %maximum workloads (*p* = 0.008), despite no differences in the average RPE reported throughout the experimental trial (*p* = 0.475).

### 3.2 Trained individuals have attenuated autophagic responses to vigorous endurance exercise, regardless of tissue type

**FIGURE 1:**
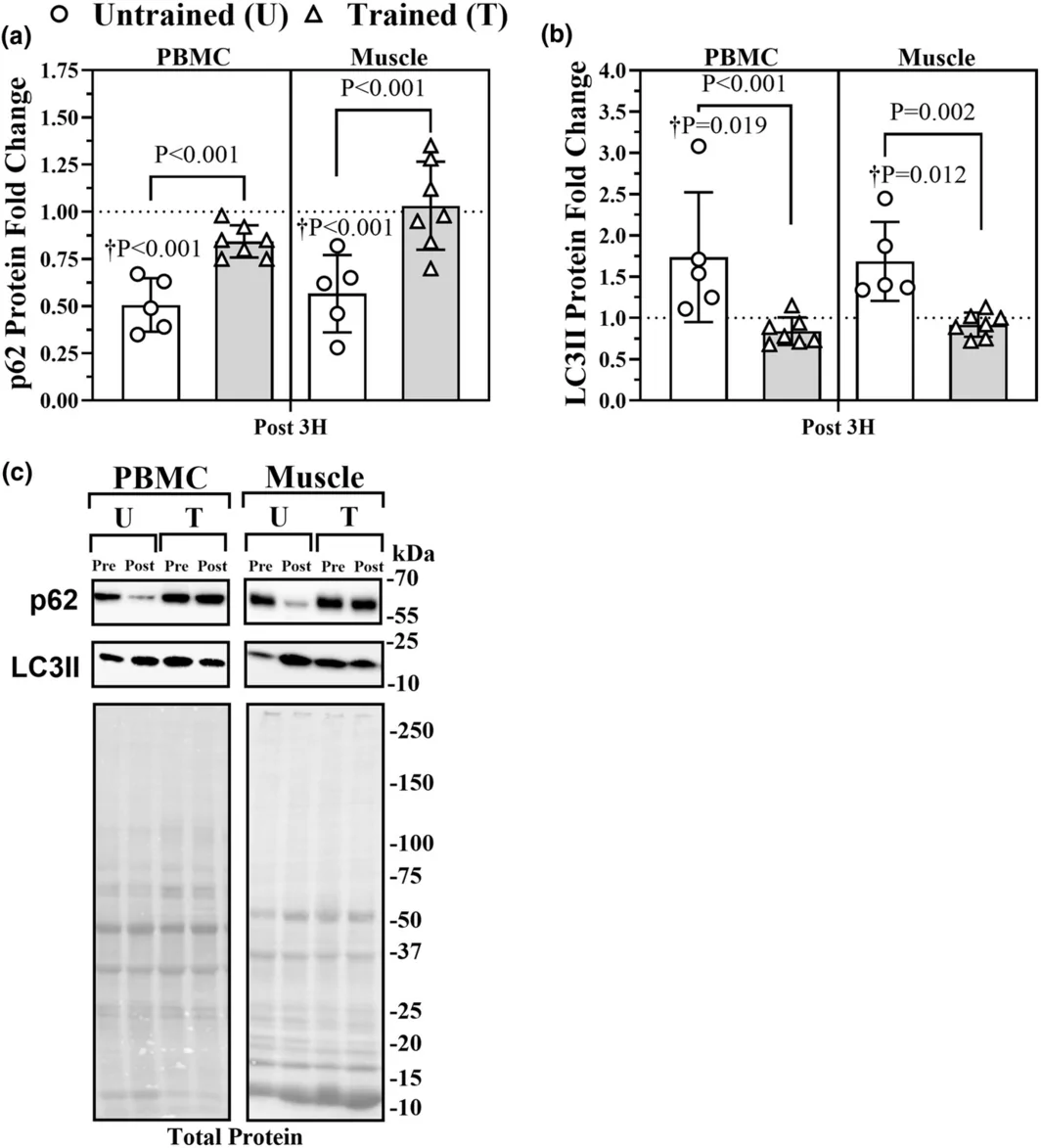
Effect of 60‐min of cycling at the second ventilatory threshold on hallmark indicators of autophagic activity (p62 and LC3II) in endurance‐trained (T) and untrained (U) men. Bar graphs represent mean ± standard deviation of the fold change compared to corresponding pre‐exercise samples (dashed lines), of p62 (a) and LC3II (b) protein in peripheral blood mononuclear cells (PBMCs) and skeletal muscle. Representative immunoblots illustrating the pre‐to‐post exercise change in p62 and LC3II within each group and tissue, and corresponding Ponceau‐stained total protein, are shown in (c). The density of proteins of interest was normalized to total protein as a loading control. Significant *p* values are presented in the figure, where † denotes *p* ≤ 0.05 compared to the corresponding pre‐exercise value with the same group and tissue. Untrained, *n* = 5; trained, *n* = 7.

Vigorous endurance exercise altered canonical autophagy‐related marker p62, as there was a main effect of time (*F*_1,10_ = 47.13, *p* < 0.001, $\eta_{p}^{2}$ = 0.85), with decreased p62 protein from pre‐ to 3 h‐post exercise (Figure 1a). While there was no main effect of tissue type (*F*_1,10_ = 3.28, *p* = 0.101, $\eta_{p}^{2}$ = 0.25), there was a main effect of training status (*F*_1,10_ = 27.32, *p* < 0.001, $\eta_{p}^{2}$ = 0.77) and time × training interaction (*F*_1,10_ = 27.32, *p* < 0.001, $\eta_{p}^{2}$ = 0.77). Post hoc analyses demonstrated that in PBMCs, protein expression for untrained participants significantly decreased from baseline (FC = 0.50 ± 0.14; *p* < 0.001), and was different (*p* < 0.001) from trained men, who did not change from baseline (FC = 0.84 ± 0.09; *p* = 0.310). The same responses were seen in skeletal muscle, as p62 protein decreased in untrained men in response to vigorous endurance exercise (FC = 0.57 ± 0.21; *p* < 0.001). This change was significantly different (*p* < 0.001) in trained individuals where p62 did not change from pre‐ to 3 h‐post exercise (FC = 1.03 ± 0.23; *p* > 0.999). Baseline p62 expression did not differ by training status, tissue type, or their interaction (all *p* > 0.05).

Similar effects of autophagy‐related signaling were seen in LC3II‐enriched signaling (Figure 1b), as there was increased protein expression from pre‐ to 3 h‐post exercise (main effect of time, *F*_1,10_ = 8.90, *p* = 0.014, $\eta_{p}^{2}$ = 0.59), with a greater response in untrained individuals (main effect of training status, *F*_1,10_ = 17.65, *p* = 0.002, $\eta_{p}^{2}$ = 0.74), and no main effect of tissue type (*F*_1,10_ = 0.01, *p* = 0.933, $\eta_{p}^{2}$ = 0.00). There was also a time × training interaction (*F*_1,10_ = 17.65, *p* = 0.002, $\eta_{p}^{2}$ = 0.74). Post hoc testing revealed that LC3II‐enriched protein response to exercise in PBMCs was different by training status (*p* < 0.001), where LC3II‐enriched signaling increased from pre‐ to 3 h‐post exercise in untrained men (FC = 1.74 ± 0.79; *p* = 0.019) but there was no change in trained participants (FC = 0.84 ± 0.17; *p* = 0.971). The same outcome was seen in skeletal muscle, as there was a different response by training status (*p* = 0.002). In untrained men, LC3II‐enriched signaling increased (FC = 1.69 ± 0.48; *p* = 0.012) but did not change in trained men (FC = 0.92 ± 0.15; *p* > 0.999). Baseline LC3II‐enriched expression did not differ by training status, tissue type, or their interaction (all *p* > 0.05).

### 3.3 Degradation of p62 is associated with increased LC3II in skeletal muscle and PBMCs

**FIGURE 2:**
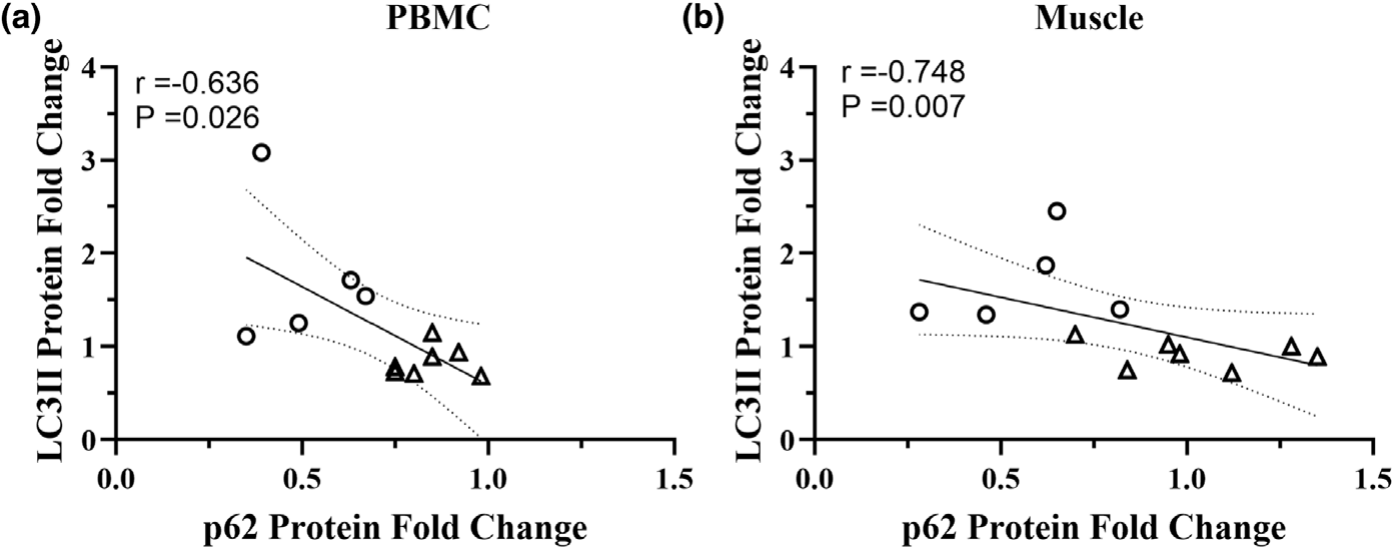
Correlations between fold changes in LC3II and p62 protein expression of endurance‐trained (triangles) and untrained (circles) men in peripheral blood mononuclear cells (PBMC; a) and skeletal muscle (b) following 1‐h of cycling at the second ventilatory threshold. Pearson's or Spearman's correlation analyses depending on the normality of the outcomes assessed along with simple linear regression analyses were used to determine relationships. The *p* value for statistical significance was set at *p* ≤ 0.05. Linear regression line with dotted lines representing the 95% confidence intervals is presented. Endurance‐trained *n* = 7, untrained *n* = 5.

To better understand the relationship between p62 and LC3II‐enriched expression as markers of autophagy in each tissue, we completed correlation analyses between change in p62 and LC3II in both PBMCs and skeletal muscle. Since the degradation of p62, indicated by a decrease in p62 protein expression, and the accumulation of LC3II, reflected by an increase in LC3II protein expression, both signal an increase in autophagy, as expected, significant negative correlations were observed between p62 and LC3II protein in both PBMC (Figure 2a, *r* = −0.636, *p* = 0.026) and skeletal muscle (Figure 2b, *r* = −0.748, *p* = 0.007) tissue samples.

## 4 DISCUSSION

The aims of our study were to determine how training status (endurance‐trained vs. untrained) and tissue type (PBMCs vs. skeletal muscle) influence the expression of markers of autophagy‐related signaling in men following an acute 1‐h bout of vigorous endurance exercise. We found evidence that exercise increased autophagy‐related markers in untrained men, as we observed decreases in p62 and increases in LC3II protein expression 3 h after vigorous endurance cycling. This response was consistent in both PBMC and skeletal muscle samples. In contrast, no change was observed in either tissue type in endurance‐trained individuals, suggesting a potential effect of training status on acute exercise‐induced autophagic activity in these tissues.

Our results demonstrate that endurance training attenuates the autophagic response to an acute bout of intense endurance exercise in men. The only previous study that we are aware of investigating the impact of exercise training on acute autophagic response to exercise had similar findings. Aas et al. (2020) demonstrated that 10 weeks of strength training in elderly (87 ± 7 years) individuals resulted in attenuated markers of autophagic flux in skeletal muscle in response to an acute bout of maximal resistance exercise compared to when the same exercise was done at baseline. The blunted response of autophagy‐related signaling in trained participants may be due to diminished returns of training, where highly‐trained individuals require a greater stimulus to induce further physiological adaptations beyond those already obtained. Additionally, these differences may reflect: (1) different temporal kinetics, where trained individuals may mount a faster autophagic response that has resolved by the 3‐h time point; (2) lower relative physiological stress at the prescribed workload, since trained participants exercised at a higher absolute wattage corresponding to their VT2 but may have experienced less cellular perturbation relative to their capacity; or (3) higher baseline autophagic tone in trained individuals, which could reduce the dynamic range of detectable marker changes. Furthermore, as excessive autophagic activity is implicated in various chronic conditions (e.g., muscle atrophy) (Sandri, 2010; Xia et al., 2021), these findings may demonstrate a therapeutic effect of regular endurance exercise by regulating autophagic responses. The single post‐exercise time point in our study precludes distinguishing among these mechanisms; however, the long‐term health consequences and mechanistic processes explaining our results warrant further research.

A notable finding from our study is that there were similar responses of markers of autophagy in PBMC's and skeletal muscle, highlighting similar responses at the local and systemic level. PBMCs are a clinically accessible, minimally invasive window into systemic cellular stress responses. Because muscle biopsy is not feasible in most clinical or population‐level settings, establishing whether PBMC autophagy markers track with skeletal muscle responses has direct translational value, particularly for monitoring autophagic dysregulation in chronic disease contexts (e.g., immunosenescence, chronic inflammation, and type 2 diabetes) where exercise is a therapeutic intervention. The acute autophagic response in PBMCs highlights the role of exercise in modulating many chronic diseases beyond just muscle diseases, including cardiovascular, neurodegenerative, metabolic, and infectious diseases (Zhou et al., 2025; Gao et al., 2025; He, Sumpter, & Levine, 2012; Kocak et al., 2022; Wu et al., 2019). To our knowledge, only one other study has compared the response of markers of autophagy between skeletal muscle and PBMCs. Escobar et al. (2021) found differential responses in PBMCs and skeletal muscle tissue after either moderate‐intensity continuous (60 min at 55% of max velocity) or high‐intensity interval (12 bouts of 1 min at 100% max velocity and 1 min at 3 miles per hour) treadmill running in active individuals. Specifically, they showed that LC3II:I ratio was altered 3‐h post‐exercise after moderate‐intensity continuous exercise in skeletal muscle but not PBMCs. Furthermore, they found a significant effect of time where p62 increased in skeletal muscle but not PBMCs for both conditions. These differences may be attributed to exercise intensity and durations, as our protocol was longer than their high‐intensity interval condition (~31 min) and more intense than the moderate intensity condition. Subsequent greater physiological strain may have led to a larger autophagic response in PBMCs in the untrained men. Additionally, Escobar et al. (2021) studied physically active individuals, not individuals of distinct training states, as in the present study, which could have potentially impacted findings.

To better understand autophagy‐related signaling, we investigated the relationship between p62 and LC3II in both tissue types, finding moderate, negative relationships in both PBMCs (*r* = −0.636) and skeletal muscle (*r* = −0.748). Although both markers are often used to indicate autophagy, they represent different points of the autophagic process. Increases in LC3II protein content are often used as evidence of autophagy, as it is a product of LC3I conjugating to phosphatidylethanolamine which occurs with the formation of the autophagosome (Burman & Ktistakis, 2010). Although LC3II formation is often associated with increased autophagic flux, it can also represent blocked degradation in lysosomes, where autophagy is decreased rather than increased (Sharifi et al., 2015). However, the combined p62 and LC3II pattern is suggestive of enhanced autophagic activity, as p62 is degraded via the autolysosome along with autophagic cargo (Liu et al., 2016).

### 4.1 Limitations

The present study possesses several limitations including limited markers of autophagy signaling and the use of a cross‐sectional study design. It is also limited in its generalizability beyond the specific populations tested. Additionally, markers of autophagy were measured at 3 h post‐exercise, and detection of autophagy‐related signaling at earlier time points may not have been observed. It is likely that the trained participants in the present study had a transient autophagic response to the exercise that occurred prior to the 3 h post‐exercise time point. For example, McCormick et al. (2022) found in habitually active males that moderate intensity exercise (55% VO_2_max) in the heat significantly increased LC3II expression in PBMCs immediately after exercise but not 3 h post‐exercise. Lastly, LC3 detection was not validated with lysosomal flux inhibitors or with antibodies that have more balanced LC3‐I/II recognition (e.g., CST #4108). Future studies will incorporate autophagic flux assays (e.g., bafilomycin A1 or chloroquine treatment) and alternative LC3 antibodies to more rigorously quantify LC3‐I/II dynamics.

## 5 CONCLUSION

To our knowledge, this is among the first studies to report a differential autophagic response to vigorous endurance exercise by training status, with results suggesting that endurance training attenuates the autophagic responses brought on by vigorous exercise. Additionally, we found similar responses between skeletal muscle and PBMC tissue types, suggesting that markers of autophagic activity in response to vigorous endurance exercise are similar at the local and systemic levels. Altogether, this study demonstrates that training status imposes differential autophagic responses to intense endurance exercise, with no differences between PBMC and skeletal muscle tissues. Although limited by sample size, we observed large time × training interaction effect sizes for p62 ($\eta_{p}^{2}$ = 0.77) and LC3II ($\eta_{p}^{2}$ = 0.74), supporting the biological relevance of the observed signal and providing important variance estimates for powering future studies. Our results from this secondary analysis provide evidence that future research is needed to further elucidate the impact which these findings have on tissue health, training adaptation, and performance.

## AUTHOR CONTRIBUTIONS

**Jonathan W. Specht:** Conceptualization; data curation; formal analysis; investigation. **Jeremy B. Ducharme:** Conceptualization; data curation; formal analysis; funding acquisition; investigation; methodology; supervision. **Alyssa R. Bailly:** Data curation. **Michael R. Deyhle:** Conceptualization; data curation; methodology; supervision.

## FUNDING INFORMATION

This research was funded by the University of New Mexico's Graduate and Professional Student Association Grant awarded to J.B.D.

## CONFLICT OF INTEREST STATEMENT

None of the authors have any conflicts of interest, financial or otherwise, to disclose.

## ETHICS STATEMENT

All procedures were approved by the University of New Mexico Institutional Review Board (Protocol Number: 2209015849) and the authors take responsibility for this work.

## Data Availability

The data that support the findings of this study are available from the corresponding author upon reasonable request.
